# Predictors of Intensive Care Unit Admissions in Patients Presenting with Coronavirus Disease 2019

**DOI:** 10.1055/s-0043-1778068

**Published:** 2024-01-31

**Authors:** Lahib Douda, Heraa Hasnat, Jennifer Schwank, Sarien Nassar, Nancy M. Jackson, Jeffrey C. Flynn, Joseph Gardiner, Dawn P. Misra, Abdulghani Sankari

**Affiliations:** 1 Department of Medical Education, Ascension Providence Hospital/Michigan State University College of Human Medicine, Southfield, Michigan, United States; 2 Department of Medicine, Michigan State University College of Human Medicine, East Lansing, Michigan, United States; 3 Department of Epidemiology and Biostatistics, Michigan State University College of Human Medicine, East Lansing, Michigan, United States; 4 Department of Medicine, Wayne State University, Detroit, Michigan, United States

**Keywords:** coronavirus disease 2019, intensive care units, mortality, risk factors, prediction model

## Abstract

**Background** Increased mortality rates among coronavirus disease 2019 (COVID-19) positive patients admitted to intensive care units (ICUs) highlight a compelling need to establish predictive criteria for ICU admissions. The aim of our study was to identify criteria for recognizing patients with COVID-19 at elevated risk for ICU admission.

**Methods** We identified patients who tested positive for COVID-19 and were hospitalized between March and May 2020. Patients' data were manually abstracted through review of electronic medical records. An ICU admission prediction model was derived from a random sample of half the patients using multivariable logistic regression. The model was validated with the remaining half of the patients using c-statistic.

**Results** We identified 1,094 patients; 204 (18.6%) were admitted to the ICU. Correlates of ICU admission were age, body mass index (BMI), quick Sequential Organ Failure Assessment (qSOFA) score, arterial oxygen saturation to fraction of inspired oxygen ratio, platelet count, and white blood cell count. The c-statistic in the derivation subset (0.798, 95% confidence interval [CI]: 0.748, 0.848) and the validation subset (0.764, 95% CI: 0.706, 0.822) showed excellent comparability. At 22% predicted probability for ICU admission, the derivation subset estimated sensitivity was 0.721, (95% CI: 0.637, 0.804) and specificity was 0.763, (95% CI: 0.722, 0.804). Our pilot predictive model identified the combination of age, BMI, qSOFA score, and oxygenation status as significant predictors for ICU admission.

**Conclusion** ICU admission among patients with COVID-19 can be predicted by age, BMI, level of hypoxia, and severity of illness.

## Introduction

The first wave of coronavirus disease 2019 (COVID-19) occurred from March to May 2020 with the number of cases peaking in April of 2020. 1 Patients who contracted COVID-19 during this time and were hospitalized had an all-cause mortality rate between 16 and 21%. 2 3 4 5 A higher all-cause mortality rate (between 35 and 42%) was reported in COVID-19 positive patients if they were admitted to intensive care units (ICUs) during this first wave of the pandemic. 6 7 8 9 Coronaviruses have a high mortality rate in critically ill patients 10 as was seen in previous severe acute respiratory syndrome coronavirus (SARS-CoV) and Middle East respiratory syndrome coronavirus. 11 Despite advanced ICU supports, the mortality rate is greater than what has been reported with previous viral pneumonitis pandemics, such as the 2009 H1N1 influenza pandemic mortality rates (35–42 vs. 5–14%). 6 7 8 12 13

The delay in ICU admission not only affects hospital resources but can impact patient outcomes both before and during the COVID-19 pandemic. 14 Conversely, an unwarranted admission to ICU can increase demand on hospital resources and lead to an insufficient availability of beds which has been linked to increased mortality from COVID-19. 15 This stark increase in mortality rates among those admitted to the ICU versus those admitted to the general floors highlights a compelling need to establish an accurate and predictive criterion for ICU admissions among COVID-19 positive patients. Current literature has already identified several clinical features associated with the severity of COVID-19 infection, and calculators have also been developed such as confusion/urea/respiratory rate/blood pressure/age>65 (CURB-65), Quick COVID-19 Severity Index (qCSI), and Brescia-COVID Respiratory Severity Scale (BCRSS) to provide a uniform analysis for ICU admission, but a simple scoring system specific to COVID-19 is lacking. 16 17 18 19 20

In this study, we aimed to identify predictors of admission to the ICU among patients admitted to the hospital with COVID-19 and develop a predictive tool for admission to ICU among these patients. We hypothesized that one or more factors relating to the severity of illness can predict which patients with COVID-19 are admitted to the ICU. In doing so, we hope to decrease ICU admission and therefore mortality in patients with COVID-19. 21

## Methods

### Participants

We conducted a retrospective observational study of patients who tested positive for COVID-19 and presented to our hospital from March 9, 2020, through May 16, 2020. Patients eligible for this study were between 18 and 99 years of age, presented to either the Southfield or Novi, Michigan campus with a diagnosis of COVID-19 determined by a nasopharyngeal swab with RT-PCR test. The study was approved by the hospital's Institutional Review Board (#1590494) prior to patient identification and data collection; a waiver of informed consent was granted due to the minimal risk nature of the study (chart review).

### Patients and Public Involvement

This was a retrospective study, and no patients were involved in the study design or in setting the research questions or reported outcomes. No patients were asked for advice on interpretation or in reporting the results.

### Data Collection

Patients' demographics, symptomatology, clinical data, laboratory results, and radiographic images were manually abstracted through review of electronic medical records by project team members. For each patient, the Charlson Comorbidity Index (CCI) was calculated by summing assigned weights to 17 comorbid conditions. 22 The quick Sequential Organ Failure Assessment (qSOFA) score was calculated from the Glasgow Coma Scale, respiratory rate, and systolic blood pressure. 23 The compiled data were de-identified and shared with a biostatistician for analysis. Data quality was ensured by random sample review by the co-investigators, continuous communication with project principal investigator and the data collection team, and by manual review of entered data by the biostatistician. Where found missing, duplicate, and discordant inputs were identified and communicated with the data collection team. They were subsequently adjusted and confirmed as appropriate. Deaths were identified by either death at discharge or death following discharge to hospice care. All discharges to hospice care during the review period were confirmed to result in death of the patient. To be conservative, we included all deaths, whether at discharge or following hospice.

### Statistical Analyses

#### Derivation and Validation Subsets

To derive a predictive model for ICU admission, we randomly split the patient cohort of *N* = 1,094 into two subsets. The first, called the derivation (or training) subset, was used to develop the predictive model from the potential correlates of ICU admission. The holdout subset, called the validation subset (also called the “test” dataset in the literature), was used solely to test the performance of the predictive model with metrics such as the c-statistic, Brier score, Hosmer–Lemeshow χ ^2^ statistic, true positive fraction, and false positive fraction. These metrics were calculated (not estimated) in the validation subset using estimated parameters from the derivation model.

It is difficult to give a general rule for the fractions of the patient cohort assigned to training and validation. Therefore, we followed a previously suggested method to split the sample at 50%. 24 The derivation subset was used to develop the prediction model for ICU admission based on demographic characteristics, vital signs, clinical and laboratory findings that were available within 24 hours of hospital admission.

Characteristics of patients were summarized as frequencies and proportions for categorical variables and by means, standard deviations for continuous variables. Comparisons between derivation and validation subsets were assessed using χ ^2^ tests for categorical variables and by Wilcoxon tests for continuous variables. Statistical significance was declared for a *p* -value <0.05.

#### Development of the Prediction Model

Multivariable logistic regression was used to construct a model for predicting the binary outcome, ICU admission, based on the variables in Table 1 . The derivation subset alone was used for this purpose. An appropriate form for continuous predictors was discerned by examining the strength of their association with outcome under different transformations. We viewed their distributions before considering the following transformations: (i) logarithm and square root, (ii) polynomial and restricted cubic spline, and (iii) categorization of the predictor to two or more levels. For example, the age at admission had a wide range, from 17 to 102 years. Its effect cannot be modeled by a single linear term because it would imply a constant risk for ICU admission at any given age. Distributions that were highly skewed required categorization. Although in some instances a more elaborate transformation such as the restricted cubic spline was more compelling, the selected form was tempered by ease of interpretability and parsimony.

**Table 1:** Characteristics of patients in full cohort, and derivation and validation subsets

Characteristics	All *N* = count (%)	Derivation *N* = count (%)	Validation *N* = count (%)	*p* -Value a
**ICU admission**				0.16 b
Yes	204 (18.6)	111 (20.3)	93 (17.0)	
No	890 (81.4)	436 (79.7)	454 (83.0)	
**Age, years**				0.04 b
< 50	210 (19.2)	92 (16.8)	118 (21.6)	
50 to <60	167 (15.3)	91 (16.6)	76 (13.9)	
60 to <70	245 (22.4)	135 (24.7)	110 (20.1)	
70 to <80	231 (21.1)	121 (22.1)	110 (20.1)	
≥80	241 (22.0)	108 (19.7)	133 (24.3)	
**Age, mean (SD)**	65.0 (17.5)	65.1 (16.5)	65.0 (18.5)	0.70 c
**Gender**				0.23 b
Female	558 (51.0)	269 (49.2)	289 (52.8)	
Male	536 (49.0)	278 (50.8)	258 (47.2)	
**Race**				0.52 b
Caucasian	278 (25.4)	131 (24.0)	147 (26.9)	
African American	785 (71.8)	404 (73.9)	381 (69.7)	
Hispanic	3 (0.3)	1 (0.18)	2 (0.37)	
Asian	15 (1.4)	6 (1.10)	9 (1.65)	
Other	13 (1.2)	5 (0.91)	8 (1.46)	
** Body mass index (BMI), kg/m ^2^**				0.015 b
< 25	272 (24.9)	126 (23.0)	146 (26.7)	
25 to <30	301 (27.5)	166 (30.4)	135 (24.7)	
30 to <35	226 (20.7)	124 (22.7)	102 (18.7)	
≥35	295 (27.0)	131 (24.0)	164 (30.0)	
**BMI, mean (SD)**	31.0 (8.4)	30.5 (7.4)	31.5 (9.2)	0.40 c
**Charlson Comorbidity Index (CCI)**				0.36 b
< 4	473 (43.2)	244 (44.6)	229 (41.9)	
≥4	621 (56.8)	303 (55.4)	318 (58.1)	
**CCI, mean (SD)**	4.3 (3.1)	4.4 (3.2)	4.2 (3.0)	0.47 c
**Systolic blood pressure, mm Hg**	129.6 (20.8)	130.2 (21.2)	129.1 (20.3)	0.42 c
Missing, count	2	2	0	–
**Diastolic blood pressure, mm Hg**	71.9 (14.0)	71.6 (14.2)	72.1 (13.8)	0.36 c
Missing, count	2	2	0	–
**Heart rate, beats/minute**	87.4 (17.5)	87.4 (17.9)	87.3 (17.1)	0.70 c
Missing, count	1	1	0	–
**Respiratory rate, breaths/minute**	20.2 (5.2)	20.3 (5.9)	20.1 (4.3)	0.85 c
Missing, count	1	1	0	–
**Temperature (°C)**	37.1 (0.7)	37.2 (0.7)	37.1 (0.7)	0.19 c
Missing, count	2	1	1	–
** White blood cell count, 10 ^9^ /L **	7.85 (4.33)	7.98 (4.36)	7.72 (4.30)	0.18 c
Missing, count	77	31	46	–
**Hemoglobin, g/dL**	12.46 (2.15)	12.41 (2.19)	12.52 (2.10)	0.41 c
Missing, count	78	31	47	–
**Blood urea nitrogen, mg/dL**	30.41 (26.70)	31.51 (28.46)	29.27 (24.73)	0.43 c
Missing, count	80	32	48	–
** Platelets count, 10 ^9^ /L **	216.23 (86.81)	213.78 (83.26)	218.77 (90.34)	0.62 c
Missing, count	79	31	48	–
** SaO _2_ /FiO _2_**				0.33 *b*
< 2	101 (9.2)	54 (9.9)	47 (8.6)	
2 to <4	321 (29.3)	169 (30.9)	152 (27.8)	
≥4	672 (61.4)	324 (59.2)	348 (63.6)	
**qSOFA**				0.32 b
0	551 (50.4)	276 (50.5)	275 (50.3)	
1	394 (36.0)	204 (37.3)	190 (34.7)	
2	132 (12.1)	57 (10.4)	75 (13.7)	
3	17 (1.6)	10 (1.8)	7 (1.3)	
**qSOFA, mean (SD)**	0.65 (0.75)	0.64 (0.74)	0.66 (0.76)	0.66 c

Abbreviations: qSOFA, Quick Sequential Organ Failure Assessment; SaO _2_ /FiO _2_ , arterial oxygen saturation to fraction of inspired oxygen ratio; SD, standard deviation.

Data presented as *N* (%) or mean (SD).

a *p* -Value for comparison between derivation and validation subsets on nonmissing data.

b *p* -Value from χ ^2^ test.

c *p* -Value from Wilcoxon test.

Log transformation was applied to white blood cell count, hemoglobin, blood urea nitrogen and platelets, whereas categories were used for age, body mass index (BMI), CCI, oxygen saturation captured by the arterial oxygen saturation to fraction of inspired oxygen (SaO _2_ /FiO _2_ ) ratio. Among the constellation of potential predictors, forward selection was applied with a liberal 15% *p* -value for variable entry. Hierarchy was required for multicategory variables. Results from the final model are presented as adjusted odds ratios (ORs) with associated 95% confidence intervals (CIs).

The CCI is a summary measure of several comorbid conditions associated with risk of mortality in hospitalized patients. Since its introduction, the CCI, and various modifications, has been used as a risk factor for outcomes other than mortality. Depending on the setting and application, one or more threshold points of the CCI have been used. 25 For our study we explored modeling the CCI (i) in its original continuous scale, (ii) as a spline function with knot placement at percentiles, and (iii) categorized at two or more thresholds as was done previously. 26

The final model was subjected to rigorous evaluation for detecting potential outliers, influential observations and was assessed for overall goodness-of-fit and predictive power. A model's predictive ability was assessed by the c-statistic, and goodness-of-fit by the Hosmer–Lemeshow χ ^2^ test, Spiegelhalter calibration test based on the Brier score (average squared error). 27 28

#### Receiver Operating Characteristic Curve

From the prediction model we obtained the patient-specific predicted probability π of ICU admission. The model's discriminative power is measured by the c-statistic. For a pair of patients, one who was admitted to ICU (case) and the other who was not admitted to ICU (control), the c-statistic is the probability that the model estimates a higher probability in the case than in the control. The c-statistic is equivalent to the area under the receiver operating characteristic (ROC) curve. For a cutoff α, the true positive fraction (sensitivity) is the proportion among cases where π ≥ α, and the false positive fraction (1 − specificity) is the proportion among controls where π ≥ α. The ROC plots the points (sensitivity, 1 − specificity) as the cut-point a varies between 0 and 1. A c-statistic above 0.75 is considered excellent. Submodels with fewer covariates may be compared with respect to their c-statistics. The true positive fraction and false positive fraction were calculated at a cutoff α = 0.22, which was near the highest point on the ROC relative to the point (0, 1) of perfect discrimination.

#### Scoring Algorithm

A total score was calculated for each patient by summation of weights assigned to the predictor variables in the final derivation model. Only the derivation subset was used for this purpose. The risk of ICU admission was assessed using the total score as a single predictor in a logistic model. Details for scoring and evaluation of the total score as a predictor are supplied in Supplementary Materials (available in the online version only).

#### Use of Validation Subset

The performance of the prediction model was validated in a dataset that had no role in model construction. Several statistics were calculated in the validation subset, including, c-statistic, Brier score, Hosmer–Lemeshow χ ^2^ statistic, true positive fraction, and false positive fraction at the same cutoff α = 0.22 used in the prediction model. 29 All statistical analyses were performed in SAS Software, version 9.4, Analytics 15.1 (SAS Institute Inc, Cary, NC).

## Results

We identified 1,094 unique patients who tested positive for COVID-19 and were admitted to our hospital between March 9, 2020 and May 16, 2020. In this cohort, 18.6% (204/1094) were admitted to the ICU.

### Demographic Characteristics

In this cohort, when Hispanic, Asian and other race are excluded, 74% (785/1,063) identified as African American (AA) and 26% (278/1063) identified as non-Hispanic White (WH). The AA group was younger on average than the WH group (mean age 64.4 ± 16.7 vs. 68.1 ± 19.1 years, *p* = 0.005), and had a higher proportion of female patients compared with the WH group (53.6 vs. 45.0%, *p* = 0.013). Mean BMI was significantly greater in AA compared with WH (31.6 ± 8.6 vs. 29.5 ± 7.8, *p* < 0.0002), with a significantly lower proportion of AA having a BMI < 25 compared with WH (22.6 vs. 32.4%, *p* = 0.001).

### Correlates of Intensive Care Unit Admission

**Fig. 1:**
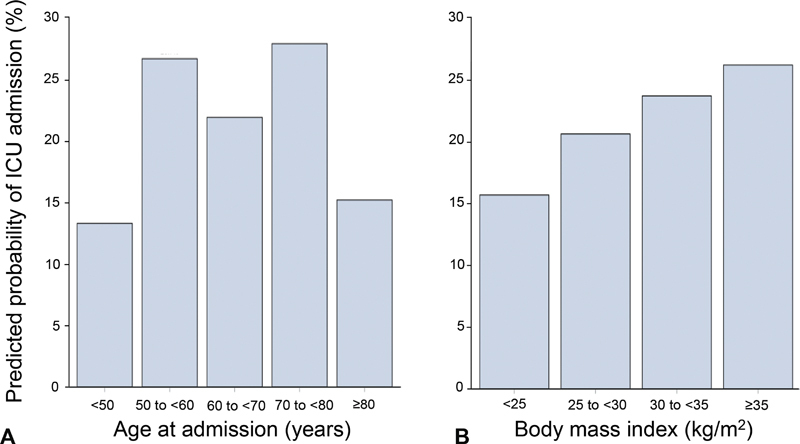
Predicted probability of ICU admission by age ( **A** ) and body mass index ( **B** ) at admission. ICU, intensive care unit.

Patient characteristics were balanced between the derivation and validation subsets ( Table 1 ). Using the derivation subset, potential correlates of ICU admission were age, BMI, qSOFA, CCI, SaO _2_ /FiO _2_ , and on the log transformed scale platelets, white blood cell count, and blood urea nitrogen. The final multivariable model derived by forward selection contained the variables in Table 2 . The SaO _2_ /FiO _2_ ratio was a strong predictor of ICU admission driven by the values <2 versus 2 to <4 (OR = 5.60, 95% CI: 2.64, 11.90). Higher qSOFA was associated with higher odds of ICU admission (OR = 2.33, 95% CI: 1.67, 3.26). A two-fold increase in platelet count was associated with an OR = 0.369 (95% CI: 0.227, 0.598). A two-fold increase in white blood cell count was associated with an OR = 1.479 (95% CI: 0.994, 2.199). Fig. 1A shows that the model predicted an average probability of 28.0% for ICU admission in the 70 to <80 age group, whereas the <50 and ≥80 age groups had lower average ICU admission probabilities at 13.3 and 15.2%, respectively. There was a gradual increase in predicted probability of ICU admission, with increasing BMI ( Fig. 1B ).

**Table 2:** Multivariable logistic regression model for intensive care unit admission: odds ratios and 95% confidence intervals

Effect	Adjusted odds ratio	95% Confidence limits		*p* -Value	Overall *p* -Value
Age, years					
** 50 to <60 versus <50**	1.636	0.659	4.059	0.288	0.006
** 60 to <70 versus <50**	1.061	0.444	2.531	0.895	
** 70 to <80 versus <50**	1.514	0.628	3.647	0.355	
** ** **≥** **80 versus <50**	0.366	0.132	1.015	0.053	
** BMI, kg/m ^2^**					
** 25 to <30 versus <25**	1.565	0.760	3.223	0.224	0.155
** 30 to <35 versus <25**	2.108	0.981	4.530	0.056	
** ≥35 versus <25**	2.243	1.061	4.743	0.034	
** qSOFA score a**	2.330	1.666	3.260	<0.0001	<0.0001
** SaO _2_ /FiO _2_**					
** ** **<** **2 versus 2 to <4**	5.602	2.638	11.897	<0.0001	<0.0001
** ≥4 versus 2 to <4**	0.737	0.428	1.269	0.271	–
** log_platelets b**	0.237	0.118	0.477	<0.0001	<0.0001
** log_WBC b**	1.758	0.991	3.118	0.054	0.054

Abbreviations: BMI, body mass index; qSOFA, Quick Sequential Organ Failure Assessment; SaO _2_ /FiO _2_ , arterial oxygen saturation to fraction of inspired oxygen ratio; WBC, white blood cell count.

a Unit increase.

b Unit increase on log scale.

### Performance Metrics

**Fig. 2:**
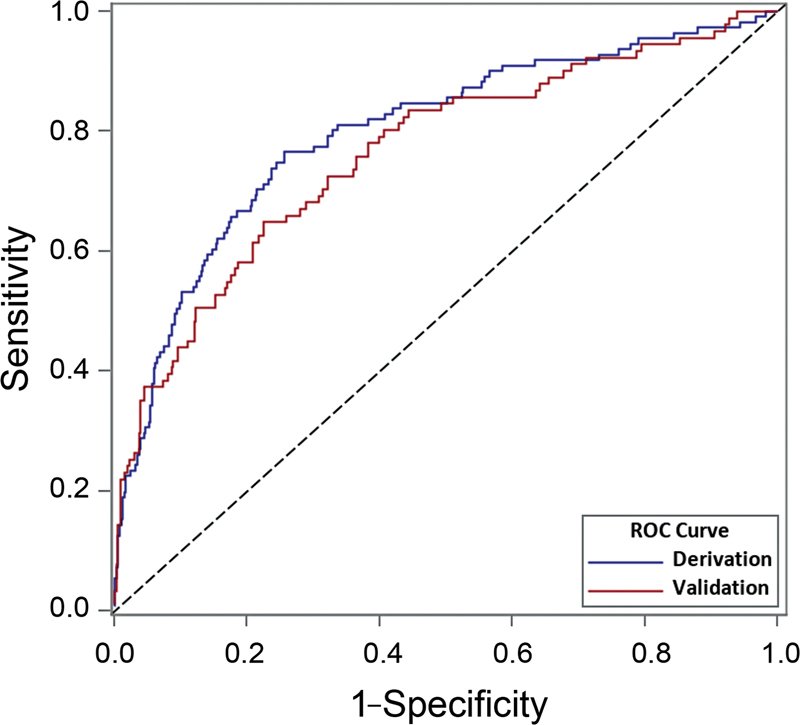
Receiver operating characteristic (ROC) curves. Derivation curve: c-statistic = 0.798, 95% confidence interval, 0.748 to 0.848. Validation curve: c-statistic = 0.764, 95% confidence interval, 0.706 to 0.822. Diagonal: Reference line.

The prediction model exhibited excellent discrimination: the c-statistic, which is the area under the ROC curve, was 0.798 (95% CI: 0.748, 0.848). The Hosmer–Lemeshow test did not indicate lack of fit ( *p* = 0.549, χ ^2^ test, 8 DF), and the Spiegelhalter calibration test based on the Brier score was also not significant ( *p* = 0.927, χ ^2^ test, 1 DF). In the validation subset, the c-statistic was 0.764 (95% CI: 0.706, 0.822) showing excellent comparability with the derivation model's c-statistic ( Fig. 2 ).

A cut-point of 0.22 in the predicted probability π( **x** ) for ICU admission was suggested by the ROC curve. Patients were classified as having the event if π( **x** ) **≥** 0.22, or not having the event if π( **x** ) **<** 0.22. As shown in Table 3 for the derivation subset, sensitivity was 0.721 (95% CI: 0.637, 0.804) and specificity was 0.763 (95% CI: 0.722, 0.804). In the validation subset, sensitivity was 0.648 (95% CI: 0.550, 0.747) and specificity was 0.762 (95% CI: 0.721, 0.804).

**Table 3:** Sensitivity and specificity: derivation and validation a

	Derivation		Validation	
Statistic	Estimate	95% Confidence limits	Estimate	95% Confidence limits
**Sensitivity**	0.7207	0.6373 to 0.8042	0.6484	0.5502 to 0.7465
**Specificity**	0.7630	0.7215 to 0.8044	0.7623	0.7209 to 0.8036
**Positive predictive value**	0.4545	0.3810 to 0.5281	0.3782	0.3021 to 0.4543
**Negative predictive value**	0.9088	0.8782 to 0.9394	0.9067	0.8759 to 0.9375

a Calculations on a predicted probability of 22% for intensive care unit admission.

### Scoring Algorithm

A simple scoring rule was obtained from the log-ORs of the six predictors in Table 2 . For each patient the “Total ICU-19” score is the weighted sum of points of values of these predictors. Details are provided in Supplementary Materials (available in the online version only). The constructed Total ICU-19 score as a single predictor of ICU admission risk had excellent performance characteristics. In the derivation subset the c-statistic was 0.771 (95% CI: 0.716, 0.825), and in the validation subset the c-statistic was 0.735 (95% CI: 0.674, 0.796).

## Discussion

The major findings of this study were that patients positive for COVID-19 with the following risk factors had an increased likelihood to be admitted to the ICU: (1) hypoxia (an SaO _2_ /FiO _2_ of <2), (2) 50 to 80 years of age, (3) morbid obesity (BMI ≥ 35), and (4) a qSOFA score ≥ 1. Thus, oxygenation status, age, BMI and qSOFA score are significant predictors for ICU admission and contribute significantly to this predictive model and scoring algorithm.

Overall, 18.6% of patients who tested positive for COVID-19 were admitted to the ICU. This is lower than the reported 32% of patients being admitted to the ICU in a large systematic review of nearly 25,000 patients. 30 The timeframe of the study and country are important as the rate of admission to ICU differed between the first and subsequent waves of the COVID-19 pandemic in 2020. Our sample was selected from the first wave of the COVID-19 pandemic for the period between March and May 2020. The latter report included studies from different countries such as China and the Middle East, and many of these reported studies did not provide data on comorbidities and risk factors for ICU admission. 31 The rate of admission to ICU in our study did not show significant differences between the derivative and validation samples. In one report from Germany, the proportion of hospitalized patients requiring ICU treatment decreased by half (from 30% early in 2020 to 14%) by the end of 2020. 32 The significant drop in admission to ICU was thought to be due to improvement in the management of patients with COVID-19 prior to requiring ICU transfer. 33

We found hypoxia indicators (SaO _2_ /FiO _2_ ) to be strongest predictors for admission to ICU, which corroborates with other studies. 34 We also found that older age (50 to <80 years) contributed to the increased risk for ICU admission; however, more than 80 years were less likely to be admitted to ICU. This finding is similar to other studies which showed that older patients were less likely to be admitted to ICU. 34 The exact cause of inverse relationship between those aged more than 80 years and ICU admission is not fully understood. It is thought to be due to an earlier presentation in older patients and changes to their code status to refuse resuscitation in-line with these patients' end-of-life goals and preferences.

Increased BMI (>35 kg/m ^2^ ) was an independent predictor of ICU admission in this study. This finding is consistent with earlier reports from Centers for Disease Control and Prevention (CDC) showing that obesity is a risk factor for hospitalization and death, particularly in younger patients (<65 years old). 35 Obesity was found also to be associated with increasing length of stay in the ICU and higher mortality. 36 However, the risk of mortality was found to be higher than those with mild to moderate obesity (BMI from 29 to 39 kg/m ^2^ ) compared with morbid obesity (BMI ≥ 40 kg/m ^2^ ) which emphasizes the importance of BMI as predictor ICU admission in the proposed model. 21

We observed that the combination of age, BMI, oxygenation, and severity of illness (i.e., qSOFA score) yielded excellent predictive performance and provided a simple and reliable diagnostic tool for predicting ICU admission among patients with COVID-19. The accuracy of the predictive model was comparable when assessed in two independent samples with high levels of concordance of statistics. Recent studies found that physiologic variables (such as heart rate, pulse oximetry, respiratory rate, and systolic blood pressure) and symptoms predicted admission to ICU. 37 Likewise, we and others found that a limited number of characteristics (age, BMI, and comorbidities) were sufficient to predict ICU admission in patients with COVID-19 and metrics of comorbidities such low oxygenation and qSOFA were the strongest predictors. 38 As reported by other studies, patients with lower oxygen saturation were more likely to be admitted to ICU as an indication of developing acute respiratory distress syndrome from COVID-19 pneumonia. 38 In contrast to earlier studies, we decided to use the ratio SaO _2_ /FiO _2_ which accurately assesses hypoxia by accounting for the level of oxygen saturation adjusted to the level of oxygen supplementation.

Our study has some limitations such as the number of patients included in this study, which was limited to one hospital system. Additionally, we focused on the first wave to avoid confounding those who received and did not receive the COVID-19 vaccination for which additional patient data could change these results; however, given the number of therapeutic options and vaccines currently available, the presence of herd immunity, different SARS-CoV-2 variants, etc., the generalizability of the results with the current state of COVID-19 may be diminished. A larger and multinational sample would be needed to address the generalizability of our findings. We have performed the study on the first surge sample during the peak of the pandemic; however, due to the limited ICU capacity and number of beds available this may have affected the threshold for ICU admission and hence the predictive model. However, validating the data internally by separate samples with similar demographics helps in ensuring the accuracy of the model. Furthermore, the changing criteria for SARS-CoV-2 testing associated with the course of the pandemic likely also affects the results.

## Conclusion

In patients with COVID-19 the combination of the dataset on age, BMI, oxygenation, and severity of illness can predict admission to ICU. The role of this model, using simple demographic and physiological data from patients recorded upon admission, in predicting clinician decision-making and patients outcomes merit additional investigation and validation.
