# Supplementary material for: Predictors of Intensive Care Unit Admissions in Patients Presenting with Coronavirus Disease 2019
Source: Avicenna J Med. 2024 Jan 31;14(1):45–53. doi: 10.1055/s-0043-1778068 (PMC11057900; doi:10.1055/s-0043-1778068)
Supplement: Supplementary file 1 — Supplementary Material [file 10-1055-s-0043-1778068-s220179.pdf]

Supplementary Materials

Scoring Algorithm

We constructed a scoring algorithm from the estimated log-odds ratios for the predictors in ►Table 2. Scores were assigned as follows: (1) for categorial variables the referent category receives a score = 0. Each other category receives a score $\beta \times 10$ where β is the regression coefficient, and 10 is a common multiplier applied to all scores for rounding. (2) The continuous variable quick Sequential Organ Failure Assessment (qSOFA) is dichotomized at zero. A qSOFA > 0 gets a score $\beta \times 10$ where β is the regression coefficient. (3) Platelets was modeled on the log-scale. Scores were assigned in three categories of the range 3.0 to 6.5 as illustrated below which applies the midpoint W and standard deviation SD. Scores are multiplied by 10 and rounded.

Log-platelets: In the range 3.0 to <5.0 midpoint $W_{REF} = 4.0$, $SD = 0.4$; score = 0.
5.0 to <6.0 midpoint $W = 5.5$; score $= \beta\,(W - W_{REF}) \times SD = -1.4393 \times (5.5 - 4.0) \times 0.4 = -0.8636$,
6.0 to <6.5 midpoint $W = 6.25$; score $= \beta\,(W - W_{REF}) \times SD = -1.4393 \times (6.25 - 4.0) \times 0.4 = -1.2954$.

Scores are multiplied by 10 and rounded.

(4) White blood cells (WBCs) were modeled on log-scale. Scores were assigned in three categories of the range −0.5 to 3.6 as illustrated below: Scores were multiplied by 10 and rounded.

Log-WBC: −0.5 to <1.4 midpoint $W_{REF} = 0.45$, $SD = 0.5$; score = 0
1.4 to <2.4 midpoint $W = 1.9$ score $= \beta\,(W - W_{REF}) \times SD = 0.5643 \times (1.90 - 0.45) \times 0.5 = 0.4091$,
2.4 to <3.6 midpoint $W = 3.0$ score $= \beta\,(W - W_{REF}) \times SD = 0.5643 \times (3.0 - 0.5) \times 0.5 = 0.7054$.

Scores are multiplied by 10 and rounded.

►Supplementary Table S1 shows the points assigned to each variable and category. For clarity, platelet count and WBC are described in their original scale. However, the scoring is unaffected. The right-most blank column will be filled for each patient. For example, the minimum total score corresponds to a patient: Age ≥ 80, body mass index (BMI) < 25, arterial oxygen saturation to fraction of inspired oxygen ratio (SaO_2/FiO_2) ≥ 4, qSOFA = 0, platelet count ≥ 400, and WBC < 4. The minimum score is $-10 + 0 - 3 + 0 - 13 + 0 = -26$.

Supplementary Table S1 Scores for predictors of intensive care unit admission

Predictor	Category range	Points	Patient score
Age, years	Below 50	0	
	50 to <60	5	
	60 to <70	1	
	70 to <80	4	
	80 or older	−10	
Body mass index, kg/m^2	Below 25	0	
	25 to <30	5	
	30 to <35	7	
	35 or higher	8	
SaO_2/FiO_2 ratio	Below 2	17	
	2 to <4	0	
	4 or higher	−3	
qSOFA	0	0	
	1, 2, or 3	9	
Platelet count, $10^9/L$	Below 150	0	
	150 to <400	−9	
	400 or higher	−13	
White blood cell count, $10^9/L$	Below 4	0	
	4 to <11	4	
	11 or higher	7	
Total score			

Abbreviation: SaO_2/FiO_2, arterial oxygen saturation to fraction of inspired oxygen ratio.

Similarly, the maximum total score (46) corresponds to a patient,

Age 50 to <60, BMI ≥ 35, $SaO_2/FiO_2 < 2$, qSOFA > 0, platelet count < 150, and WBC ≥ 11.

The observed range of total score was −22 to 43 in both the derivation and validation subsets.

 © 2024. The Author(s).

Characteristics of Total Score

Supplementary Table S2 Frequency and proportion of patients admitted to intensive care unit by total score in derivation subset

Total Score	<5	5 to <12	12 to <19	≥19
Number of patients admitted in ICU	20	26	21	44
Total number of patients	234	143	77	62
Percentage of patients admitted in ICU	8.6	18.2	27.3	71.0

Abbreviation: ICU, intensive care unit.

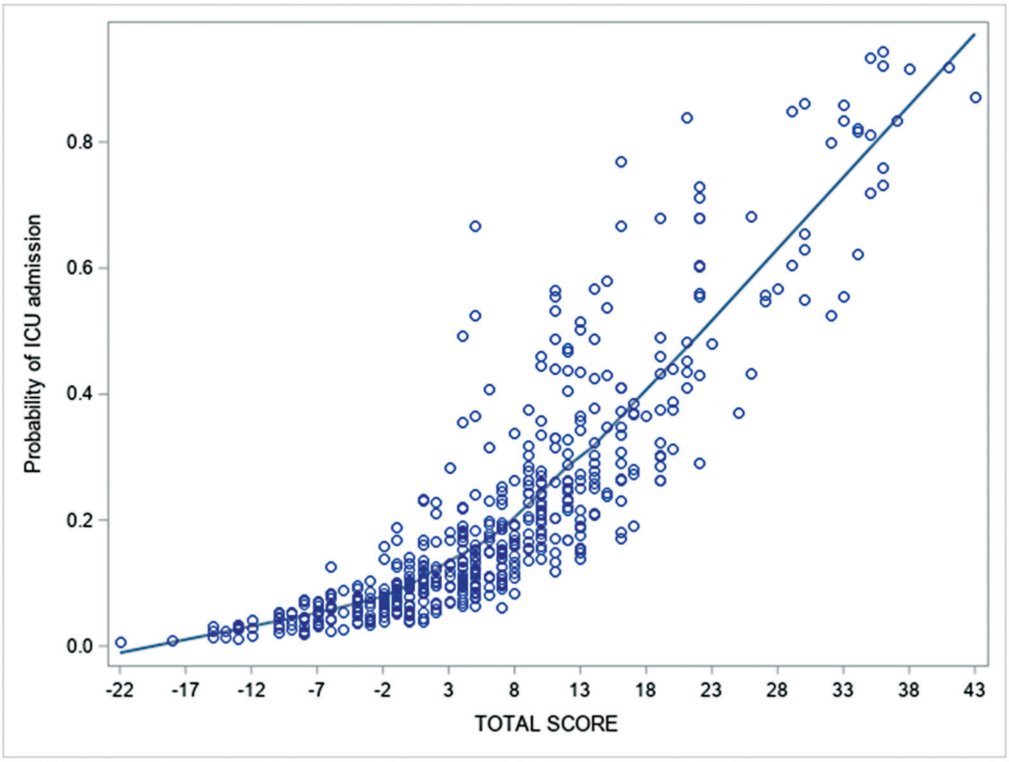

Supplementary Fig. S1 Predicted probability of ICU admission in the derivation subset by Total Score as predictor. Smoothed curve obtained by Loess smoothing of predicted probabilities (open circles) of ICU admission from logistic regression with Total Score as predictor. C-statistic=0.735, 95% confidence interval, 0.674 to 0.796 in the validation subset. ICU, intensive care unit.

References

1 Steyerberg EW. Clinical Prediction Rules, 2nd ed. Switzerland:: Springer-Nature;; 2019
2 Foieni F, Sala G, Mognarelli JG, et al. Derivation and validation of the clinical prediction model for COVID-19. Intern Emerg Med 2020;15(08):1409–1414
3 Gude-Sampedro F, Fernández-Merino C, Ferreiro L, et al. Development and validation of a prognostic model based on comorbidities to predict COVID-19 severity: a population-based study. Int J Epidemiol 2021;50(01):64–74
4 Han K, Song K, Choi BW. How to develop, validate, and compare clinical prediction models involving radiological parameters: study design and statistical methods. Korean J Radiol 2016;17(03):339–350

 © 2024. The Author(s).
